# Moral Identity and Attitudes towards Doping in Sport: Whether Perception of Fair Play Matters

**DOI:** 10.3390/ijerph182111531

**Published:** 2021-11-02

**Authors:** Saulius Sukys, Ilona Tilindiene, Daiva Majauskiene, Diana Karanauskiene

**Affiliations:** Department of Physical and Social Education, Lithuanian Sports University, Sporto 6, LT-44221 Kaunas, Lithuania; ilona.tilindiene@lsu.lt (I.T.); daiva.majauskiene@vdu.lt (D.M.); diana.karanauskiene@lsu.lt (D.K.)

**Keywords:** moral identity, fair play, attitudes towards doping, athletes and non-athlete students

## Abstract

Research evidence suggests that athletes’ attitudes towards banned substances are among the strongest predictors of intention to use or actual practice of doping. Previous research has found that personal morality was negatively related to doping attitudes. However, less is known about the role of athletes’ perceptions of fair play on their attitudes towards doping. First, we examined whether moral identity was associated with athletes’ attitudes towards doping and whether their perceptions of fair play mediated this relationship. The second purpose was to determine whether these associations differed among non-athletes. Overall, 365 university students (49.9% males, 55.3% athletes) participated in this study (mean age 22.02, SD = 6.58). They completed questionnaires measuring the aforementioned variables. The results showed that athletes’ moral identity and endorsement of fair play were negatively associated with their attitudes towards doping. The mediation analyses showed that the effect of moral identity on attitudes towards doping was partially mediated by perceptions of fair play (indirect effect, β = −0.10, *p* < 0.05). Unlike student athletes, non-athletes’ moral identity negatively predicted attitudes towards doping only indirectly, via fair play perception (indirect effect, β = −0.08, *p* < 0.05). The study provides insights into how a person’s morality and perception of moral values in sport may act as factors related to doping in sport. The practical implications for the promotion of anti-doping attitudes for athletes and separately for student non-athletes were provided together with future research perspectives.

## 1. Introduction

Sport has to provide opportunities for athletes to compete and thus demonstrate their skills in fair play [1,2]. However, in contemporary highly competitive sport, moral norms are often overlooked for other more selfish interests, such as winning at any cost [3]. Therefore, some athletes not only try to put effort into improving their mastery in order to succeed, but at the same time take risks by using banned performance-enhancing drugs or methods referred to as doping. Some studies have revealed that up to 57% of elite athletes use doping for performance improvement [4]. World Anti-Doping Agency doping control tests showed that different groups of banned substances are disproportionately spread in different sports disciplines but doping itself is used in both individual and team sports [5]. Although efforts are being made to test athletes more, this has not yet yielded significant results [6]. Therefore, in order to develop and improve doping prevention programs, it is important to have a good understanding of the factors that influence athletes’ choices to use doping in sport [7,8].

Research suggests that various personal and psychosocial factors influence athletes’ choices to use doping [9,10], but the attitudes of athletes play a special role. The evidence suggests that attitudes are reliable predictors of behavior [11]. A meta-analysis of Ntoumanis et al. [10] and Blank et al. [9] found that athletes’ attitudes towards banned substances were among the strongest predictors of intention to use or actual practice of doping. Besides, a recent study by Nicholls et al. [12] also suggested that athletes’ more positive attitudes towards doping were related to cheating behaviors. Therefore, understanding the factors that influence a person’s attitudes towards doping is important.

To date, much is already known about personal and social contextual and personal factors influencing athletes’ attitudes towards doping. Considering the social context, evidence suggests that the people surrounding the athlete (especially the coach) are important in shaping athletes’ attitudes towards doping. Trust, a respect-based relationship between the coach and athletes [13,14], the coach endorsing anti-doping attitudes [14] as well as a secure attachment to the coach and teammates influence anti-doping attitudes [15]. Greater perceived social pressure to engage in doping [16], athletes’ contacts with doping users also related to more positive attitude towards doping [17]. Thus, the environments in the team and its standards are of vital importance [18] likewise sports culture [9].

However, personal factors are also important. Several studies have revealed positive associations between extrinsic motivation and athletes’ attitudes towards doping [17,19,20]. Attitudes towards doping are also related to personality traits. Previous studies found that athletes with extremely high perfectionism levels were more inclined towards doping [17,21]. In addition, it was found that striving for perfectionism negatively predicted, while perfectionism concerns positively predicted athletes’ attitudes towards doping [20,22]. Athletes’ perceptions of competence and success also are important variables. As evidence suggests, task orientation was negatively related, and ego orientation was positively related, to attitudes towards the use of doping [18,22]. It is also important to understand how the essential moral principles or moral values in sport are perceived. How athletes understand and respect the rules, rituals, and traditions of sport and are able to distinguish what is good and bad is associated with the concept of sportsmanship [23], which is very close to the definition of fair play that represents what is morally right and characterizes good sporting competition [24]. Some studies showed that sportsmanship orientation is negatively related to the intention to use doping [16]. So, how a person perceives what is morally right in a sport may reflect certain personal values, and in turn, personal attitudes may reflect the expression of certain values. As previous research has revealed, moral values of athletes in sport activities may be negatively related to their attitudes to deception [25]. Therefore, the perception of fair play as an expression of what is morally right in sport is an important variable and might affect attitudes to doping in sport, which has so far received insufficient attention in research.

The use of banned performance-enhancing substances in sport is associated with a moral choice, a choice based on principles of right and wrong [26]. Thus, personal morality, among other factors, is important [26]. Some scholars indicate that personal morality might be the most important influencing variable in doping attitudes [27,28]. Based on a social cognitive theory [29], individuals develop moral standards that govern their behaviors. People’s actions depend on their moral standards and therefore we are personally responsible for our actions. Emotions are also a very important factor in regulating moral actions. It has been found that unpleasant emotional consequences such as self-condemnation and guilt can help an athlete refrain from using prohibited substances [30,31]. However, sometimes people behave immorally, violating their personal moral standards without self-sanction via the use of moral disengagement [29]. It is noteworthy that many studies have been conducted recently that have revealed that moral disengagement is directly or indirectly related to doping attitudes [32,33] and is especially related to the likelihood or intention to use doping [34,35,36].

Another important factor related to moral behavior is moral identity. Aquino and Reed [37] defined moral identity as a self-regulatory mechanism. Specifically, this construct refers to a cognitive schema that people hold about their moral character and reflects the importance that one places on being a moral person [37]. Moral identity can help to maintain a balance between how we perceive ourselves as a moral self and our actions [38]. Therefore, a strong moral identity can motivate people to act morally [38]. In addition, they do not have to activate moral disengagement mechanisms to suppress the effects of negative emotions associated with unethical behavior [34]. We suppose this construct is also very important as it is beyond sport, i.e., moral identity describes the degree to which being a moral person is central to one’s self-concept, not just a moral person as an athlete [37,39]. However, the ways that the moral identity of athletes is related to doping behavior have been more actively addressed only in recent years. A qualitative study by Erickson et al. [13] showed that a strong moral stance was important as a protective factor against doping in sport. Other quantitative studies confirmed that moral identity negatively predicted athletes’ doping likelihood [34,35,40]. Stanger and Backhouse’s [36] results also showed that athletes with a stronger moral identity were less likely to use a banned substance even if they were more susceptible to justify doping. These significant studies provided evidence that moral identity was an important factor in analyzing doping issues. Therefore, based on the findings of these studies, it is possible to suppose that athletes with a stronger moral identity may have less-positive attitudes towards doping. However, research has not yet addressed this possibility.

### The Present Study

Research evidence suggests that various contextual and personal factors are related to athletes’ attitudes towards doping [13,14,15,16,17,18,19,20,21,22]. In this study, we focus more on personal factors, the role of which in doping attitudes is less clear. Specifically, less attention has been paid to the role of moral identity in predicting athletes’ attitudes towards doping. Furthermore, so far, there are contradictory data regarding the perceptions of moral principles in sport as well as values related to the attitudes of athletes towards doping, especially among adult athletes. This is important as it will complement existing data on the importance of moral identity in understanding doping-related behaviors, thus it will also help to better understand the importance of athletes ’perceptions of moral principles in our study—the perception of fair play. Therefore, the purpose of our study was to examine whether moral identity was associated with athletes’ attitudes toward doping and whether perceptions of fair play mediated this relationship. Based on previous findings on the likelihood to use doping [34,35,40], we hypothesized that moral identity would be inversely associated with attitudes towards doping. As perceived moral values in sport can negatively predict attitudes on cheating behavior [25], we also expected that an endorsement of fair play, as an expression of moral principles in sport, would be negatively associated with doping attitudes. Finally, we hypothesized that the perception of fair play would mediate the relationship between moral identity and attitudes towards doping.

Researchers [34] note that most studies examining the association between moral identity and doping were conducted with British athletes, thus it is relevant to continue such research with participants from other countries. In this sense, our study contributes to this call. However, in our study, we also want to point out that researchers have not examined how moral identity is related to attitudes towards doping in sport among non-athletes, thus whether our hypotheses will also be supported. A better understanding of how doping is perceived and valued not only by athletes but also by people not participating in sport is important for an effective anti-doping strategy [41]. Not only modern testing tools and financial resources for athletes, but also public support, are important in the fight against doping. Research with adults shows that most of them are against doping in sport [41,42]. Longitudinal observations have also shown that people, like athletes, tend to see doping in sport as a more serious problem [41]. However, observing the change in students’ attitudes during the study year, it was observed that there was a tendency to support the internationally promoted “zero tolerance” policy less, and students became more tolerant of using doping [43]. In addition, in the evaluations of doping, there may be a conflict of values where the sports achievements of athletes of a favorite team or country are seen as more important than how fairly they achieved it. People who are very interested in sport have been found to have more liberal attitudes towards doping and related scandals [44]. In conclusion, research on non-athletes’ attitudes towards doping in sport is also relevant, especially if it is population-based. Our study covers only students and will not be a population-based study of youth attitudes. However, it will provide an answer to the second aim of the study—to establish whether the moral identity of students who do not participate in sports and endorse fair play is associated with attitudes towards doping as they are among student athletes.

## 2. Materials and Methods

### 2.1. Participants

Participants in this study were 365 university students (49.9% male) recruited from Universities in Lithuania. At the time of data collection, participants ranged in age from 18 to 30 years (mean age 22.02, SD = 6.58). In the sample, 55.3% (n = 202, 65.3% male) were athletes who competed in individual (n = 118, 50.0% male) and team sports (n = 84). The individual sports included swimming, tennis, table tennis, cycling, athletics, boxing, wrestling, and judo. The team sports included basketball, football, handball, and volleyball. At the time of data collection, participants had competed in their sport for an average of 8.70 (SD = 4.32) years. Among athletes, 32.2% (n = 65) were currently competing or had recently competed at the international level, 46.0% (n = 93) at the national, and 21.8% (n = 44) at the regional or university level.

### 2.2. Measures

#### 2.2.1. Doping Attitudes

The 8-item version of the Performance Enhancement Attitude Scale [45] was used in this study. The original scale consisted of 17 items [8], but Nicholls et al. [45] found a better fit of an 8-item scale within a sample of adult athletes. The Lithuanian version of this scale also resulted in a better fit of an 8-item PEAS [46]. Therefore, participants were asked to indicate their level of agreement with eight statements (e.g., “Legalizing performance enhancements would be beneficial for sports”, “Athletes should not feel guilty about breaking the rules and taking performance-enhancing drugs”, “Doping is necessary to be competitive”) using a Likert scale, ranging from strongly disagree (1) to strongly agree (6). The mean of the eight item ratings was computed and used as a measure of attitudes to doping. The internal consistency of the scale scores in this study was good (α = 0.81).

#### 2.2.2. Moral Identity

In this study, we used the internalization dimension of the moral identity scale [37] to measure moral identity. Participants were presented with nine traits (fair, honest, helpful, kind, generous, compassionate, caring, fair, friendly, and hardworking) related to common characteristics of moral individuals and asked to imagine how a person with the given traits would feel, act, and think, and to respond to five statements (e.g., “It would make me feel good to be a person who has these characteristics”, “Being someone who has these characteristics is an important part of who I am”, “I strongly desire to have these characteristics”) on a 7-point Likert scale, ranging from strongly disagree (1) to strongly agree (7). The mean of the five item ratings was used as a measure of moral identity. The internal consistency of the scale scores in this study was good (α = 0.73).

#### 2.2.3. Perception of Fair Play

The perception of fair play was measured using the Fair Play scale [47]. Participants were given 10 items (e.g., “It is impossible to do well in sports if you play fair”, “You can win playing fair”, “In sports it is acceptable that one tries to bend the rules”). They had to rate each item on a 4-point Likert scale, ranging from “strongly agree” to “strongly disagree”. Some items were rated in reverse scores. Based on Majauskiene’s [48] study, we used this scale as a unidimensional scale. A higher overall score demonstrated a higher endorsement of fair play. The Internal consistency of the scale scores in this study was good (α = 0.77).

### 2.3. Procedure

Before the study, approval from the university social research ethics committee was obtained. Oral informed consent was obtained from all research participants. All participants were informed about the research aim, study duration, risk and benefits, and the right to refuse to participate or withdraw from the survey. Students did not have to disclose any personal information (e.g., names, dates of birth, study program, contact details) and were told that all data would be kept anonymous and the information they provided would be used only for research purposes. Those who volunteered to participate were instructed how to complete the measures described above. This whole procedure was performed in a university auditorium at the end of a lecture or a seminar.

### 2.4. Statistical Analysis

The data obtained through the survey were exported into an SPSS data file and analyzed using the IBM SPSS version 26 package. Before the main statistical analyses, preliminary data screening was conducted in order to check for data normality, missing values, and outliers for each variable. It was found that 0.3% of the data points were missing and were replaced with the mean of the respective variable. Analyses indicated that that skewness and kurtosis for all variables were low (i.e., ≤1). Descriptive statistics (i.e., mean, standard deviation, and Pearson’s correlations) were calculated for all variables. Reliability estimates were calculated for all variables using alpha coefficients. Scores of all variables showed acceptable internal consistency values. Comparisons of study variables between athletes and non-athletes were conducted using one-way ANOVA. Mediation analyses were performed using the PROCESS 2.16 [49] SPSS macro (model 4) aiming to test direct and indirect effects. Direct effects are the effects of the predictor on the outcome variable that occur separately to the mediator, while indirect effects are the effects of the predictor on the outcome variable via the mediator. Bootstrapping was set at 10,000 samples, and bias-corrected 95% confidence intervals were calculated for all effects. An effect is significant when the CI does not contain zero. The completely standardized indirect effect (CSIE) was reported as the effect size metric and interpreted as 0.01 = small effect, 0.09 = medium effect, and 0.25 = large effect [50].

## 3. Results

### 3.1. Descriptive Statistics and Correlations

Study results showed that participants could be characterized by a relatively high moral identity, they relatively endorsed fair play, and had negative attitudes to doping in sport (Table 1). Correlations indicated that moral identity was negatively associated with positive attitudes to doping and positively associated with an endorsement of fair play. The fair play variable was also negatively associated with positive attitudes towards doping.

### 3.2. Comparison between Athletes and Non-Athletes

A one-way ANOVA showed that athletes (*M* = 1.53, *SD* = 0.60), compared to non-athletes (*M* = 1.40, *SD* = 0.46), had significantly more positive attitudes towards doping (*F*(1, 363) = 5.32, *p* < 0.05, partial η^2^ = 0.01). However, non-athletes (*M* = 3.13, *SD* = 0.42), compared to athletes (*M* = 3.02, *SD* = 0.38), demonstrated more positive perceptions of fair play (*F* (1, 363) = 7,26, *p* < 0.01, partial η^2^ = 0.02). When comparing moral identity, a statistically significant difference was not found (*F*(1, 363) = 3,48, *p* > 0.05).

### 3.3. Main Analysis

**Figure 1:**
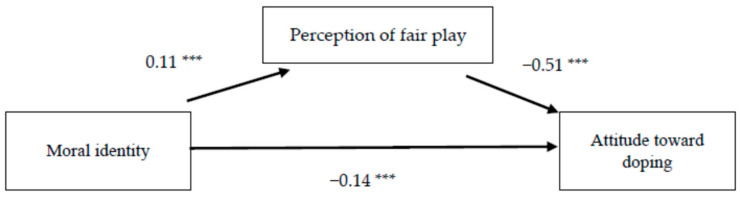
The effects of moral identity on attitudes to doping and the mediating role of perception of fair play among athletes. Note: The values presented are the unstandardized regression coefficients. A solid line represents a significant relationship. *** *p* < 0.001.

First, we investigated whether moral identity was associated with athletes’ perception of fair play and attitudes towards doping in sport, and whether the effect of moral identity on attitudes to doping was mediated by perception of fair play. It was found that moral identity had significant direct effects on attitudes towards doping (β = −0.14, *p* < 0.001) and a significant indirect effect via endorsement of fair play on attitudes to doping (β = −0.10, *p* < 0.05) (Table 2 and Figure 1). The more positive perceptions for fair play that were demonstrated were also significantly related to attitudes to doping (β = −0.51, *p* < 0.001. These findings provide support for the mediating role of endorsement of fair play on the relationship between moral identity and attitudes to doping (*F* = 25.12, *p* < 0.001, *R* = 0.45).

**Figure 2:**
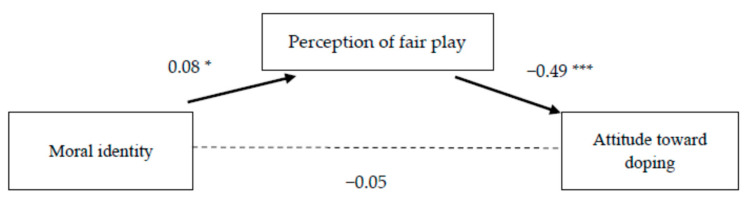
The effects of moral identity on attitudes to doping and the mediating role of perception of fair play among non-athletes. Note: The values presented are the unstandardized regression coefficients. A solid line represents a significant relationship. * *p* < 0.05; *** *p* < 0.001.

Next, we investigated whether the moral identity of non-athletes was associated with their perception of fair play and attitudes towards doping in sport, and whether the effect of moral identity on attitudes to doping was mediated by the perception of fair play. Analyses showed that the moral identity of non-athlete students was not directly related to attitudes towards doping (Table 3 and Figure 2). Results of the analysis show that moral identity was directly positively related to participants’ endorsement of fair play (β = 0.08, *p* < 0.05). Importantly, moral identity had a significant indirect effect on attitudes towards doping via the perception of fair play (β = −0.08, *p* < 0.05) (*F* = 18.16, *p* < 0.001, *R* = 0.43).

## 4. Discussion

It has been proposed that personal morality might be the most influencing variable on doping attitudes [27,28]. Building on research conducted on the role of moral identity on doping likelihood [34,35,40], we examined the association between student athletes’ moral identity and attitudes towards doping, and whether their perception of fair play mediated this association. Next, we analyzed the same relationship among non-athlete students. The study provided evidence on these relationships and at the same time allowed for practical guidance to be provided separately for athletes and non-athlete students.

### 4.1. Athletes’ Moral Identity, Perception of Fair Play and Attitudes towards Doping

In support of our hypothesis, we found that moral identity was negatively associated with athletes’ attitudes towards doping. This result is in line with existing cross-sectional studies [28] reporting that elite Australian athletes with a weaker moral stance against the use of performance-enhancing substances had more favorable attitudes towards doping. However, it should be noted that in these previous studies, morality was measured as a judgment of cheating or as a moral judgment of doping. In our study, we assessed morality not as a specific action precisely in the context of sport. Rather, our focus was on moral identity, which reveals how morality is important in personal self-perception. Therefore, our finding extends past works by revealing that using doping is viewed as unethical behavior, which is not compatible with the perception of the athlete as a moral person.

Emphasizing the importance of moral values in sport, this study also examined the relationship of the mediating role of the perception of fair play in moral identity and attitudes towards doping. The study data confirmed that endorsement of fair play mediated the relationship between athletes’ moral identity and attitudes towards doping. Thus, athletes with a stronger moral identity may perceive fair play in sport as more important and, as a result, have more negative attitudes towards doping. On the other hand, those athletes showing less respect to fair play may have lower moral standards and thus demonstrate more positive doping attitudes. The current finding is also important because the perception of fair play reveals the individual’s value orientations in sports-related behavior. It is values that shape personal attitudes; in other words, attitudes are characterized by the function of expressing values [51]. Therefore, when assessing the impact of attitudes on behavioral decisions and behavior, the orientation of the athlete to moral values in sport is also important. Previous studies with adolescents and adult athletes [16,52] revealed not only a correlation between perceived moral values and attitudes to doping, but also that attitudes fully mediated the effect of sportspersonship orientation on doping intention.

The athletes’ study results provide rationale that strengthening the moral identity of athletes and, at the same time, promoting their anti-doping attitudes and internalization of moral values is important, which is in line with other research. A recent randomized control trial provides support that intervention not only involves knowledge about doping, but that moral values in sport also affect the moral identity of athletes [53]. On the other hand, it is not enough to limit oneself to sport-related values or to emphasize their importance in sport alone, especially in strengthening moral identity. Ring et al. [54] examined the relationships between Schwartz’s basic values and doping likelihood among university athletes and found that self-enhancement values were positively related, whereas self-transcendence and conservation values were negatively related, to doping likelihood. Therefore, in order to strengthen the moral identity of athletes and promote more negative attitudes to doping in sport, coaches need to not only encourage athletes to analyze situations related to both doping specifically and anti-social behavior in general. In this debate, it is also important to promote personal responsibility in situations that raise ethical dilemmas. Strengthening athletes’ moral identity requires the integration of the moral values of sport with personal values. In other words, the analysis of the compatibility of personal values with those that are important in sport, or the existence of certain contradictions in values, should be encouraged. It also requires the knowledge of the values of athletes that are the most important for them as moral persons and as athletes. Often, various doping prevention recommendations emphasize the role of the coach as a key person in the team. However, in implementing the recommendations made in the context of adult sport, a sports psychologist, if available, can also significantly help.

### 4.2. Non-Athletes’ Moral Identity, Perception of Fair Play, and Attitudes towards Doping

In this study, we also aimed to measure the relationship between moral identity, perception of fair play, and attitudes among non-athlete students. In other words, we sought to determine if the established relationship between the study variables among athletes would differ, and if it differed, to what extent. To our knowledge, this is the first study examining such relationships among non-athletes, except for studies comparing athletes and non-athletes’ attitudes towards doping [41,55]. Before discussing the main findings it should be noted that our study found athletes’ attitudes towards doping to be more positive compared to those of non-athletes. This is not consistent with other studies, which have demonstrated that athletes hold more negative attitudes than the general population [41,55]. However, research participants’ age and time of the study as well as the cultural context need to be considered when comparing the data. Therefore, we will not further analyze these differences and rather focus on the main results. It should be acknowledged that moral identity was not directly associated with non-athletes’ attitudes towards doping. However, higher endorsement of fair play was negatively related to doping attitudes. Furthermore, we found that the perception of fair play mediated the relationship between moral identity and doping attitudes, suggesting that if people with a stronger moral identity are also more likely to endorse fair play, they would demonstrate more negative attitudes towards doping. It should be acknowledged that the main difference between non-athletes and athletes in our study lies in the direct effect of moral identity on attitudes. Interestingly, the effect of the perception of fair play on doping attitudes is the same among both athletes and non-athletes. These findings highlighted that in people’s moral schema, there may be certain behavior that would be morally wrong in everyday life but perceived as not so wrong in the sports context, as something separate from everyday life. In other words, people may view doping as a minor problem as it is related to a limited number of athletes [41], and such behavior is related to athletes’ morality [56]. However, we would still assume that how a person values moral values and moral behavior in sports is important. As our data revealed, if fair play is perceived as important in sport, people with a stronger moral identity tend to evaluate morally wrong behavior in sport more negatively. Thus, our data partially extend previous research suggesting that non-athletes may experience a potential conflict of values when evaluating what is good and bad behavior in sport [41,56], especially regarding their interest in sport [44].

Our findings on the relationship between non-athletes’ moral identity, the perception of fair play, and attitudes towards doping have some practical implications. As university students were involved in our study, in their study modules, it would be useful to include topics on the use of banned drugs in sport or on cheating in sport in general as morally inadequate behaviors. Moreover, it is necessary to communicate and discuss that such behavior is morally wrong not only in the sports context. In this way, their attitudes towards doping as an essentially moral problem could be encouraged. Students’ negative attitudes towards this are important as some of these students will occupy various sport-related decision-making positions in their future professional lives. If their professional activities are not related to sports, their moral position as citizens would remain important.

### 4.3. Limitations and Future Research

This study is not without limitation. The study analyzed the attitudes towards doping only and did not include actual behaviors. However, this was partly due to the inclusion of non-athlete subjects in the study whose actual doping behavior could not be investigated. It would be worthwhile to include a variable of intent to use banned drugs in further research. Another limitation of the study is related to the relatively small sample size. A larger sample of athletes, especially involving those in a wider variety of sports branches, would allow one to examine the extent to which the interrelationships between moral identities, perception of fair play, and doping attitudes occur depending on the sport. This claim corresponds with recent research that attempted to analyze attitudes and views on doping in particular sports such as track and field [57] or, more specifically, elite distance running [58]. Such analyses are also encouraged by doping control test findings showing that detected banned substances in anti-doping control tests differ depending on the sports discipline [5].

As this study exclusively examines athletes who are studying at university, it would be worthwhile to examine non-students in sport or those athletes who have already completed their studies. It should also be mentioned that there is still a great lack of longitudinal studies to better understand how athletes’ perceptions of moral values and attitudes towards them, as well as their real behaviors, change. In the study of non-athletes, population surveys remain relevant to reveal how people evaluate the doping problem in sport. Research that allows comparisons to be made of how doping evaluations vary depending on different cultural contexts should also be encouraged.

## 5. Conclusions

In conclusion, the current study reinforces the assertion that both moral identity and perception of fair play are important constructs affecting athletes’ attitudes towards doping. Our findings suggest that those with a stronger moral identity and higher endorsement of fair play demonstrate more negative attitudes towards doping. The study revealed that the evaluation of fair play is a factor directly related to non-athletes’ attitudes, and moral identity as a single factor is not related to students’ attitudes. This suggests that non-athletes perceive moral issues in sport as possibly more related to the context of sport and less as generally morally wrong behaviors.

## Figures and Tables

**Table 1:** Descriptive statistics and correlations.

	M	SD	α	1	2	**3**
1. Moral identity	6.05	0.92	0.73			
2. Perception of fair play	3.07	0.40	0.77	0.24 **		
3. Attitudes towards doping	1.47	0.55	0.81	−0.23 **	−0.41 **	

Note. ** *p* < 0.01.

**Table 2:** Direct and indirect effects of moral identity on attitudes to doping among athletes.

Pathways	β	95% CI	CSIE	95% CI
Direct effects of moral identity on				
Attitude to doping	−0.14 ***	[−0.21. −0.06]		
Perception of fair play	0.11 ***	[0.05. 0.16]		
Direct effect of perception of fair play on				
Attitude to doping	−0.51 ***	[−0.73. −0.32]		
Indirect effect on attitudes to doping via				
Perception of fair play	−0.10 *	[−0.16. −0.04]	−0.09 *	[−0.17. −0.04]

Note: Unstandardized coefficients for the paths are shown. CSIE: completely standardized indirect effect, where 0.01 = small, 0.09 = medium and 0.25 = large. * *p* < 0.05; *** *p* < 0.001.

**Table 3:** Direct and indirect effects of moral identity on attitudes to doping among non-athletes.

Pathways	β	95% CI	CSIE	95% CI
Direct effects of moral identity on				
Attitude to doping	−0.05	[−0.11. 0.06]		
Perception of fair play	0.08 *	[0.01. 0.16]		
Direct effect of perception of fair play on				
Attitude to doping	−0.49 ***	[−0.65. −0.33]		
Indirect effect on attitudes to doping via				
Perception of fair play	−0.08 *	[−0.17. −0.01]	−0.07 *	[−0.15. −0.01]

Note: Unstandardized coefficients for the paths are shown. CSIE: completely standardized indirect effect, where 0.01 = small, 0.09 = medium and 0.25 = large. * *p* < 0.05; *** *p* < 0.001.

## Data Availability

The data presented in this study are available on request from the corresponding author.

## References

[1] Serrano-Durá J., Molina P., Martínez-Baena A. (2021). Systematic review of research on fair play and sporting competition. Sport Educ. Soc..

[2] Molina P., Úbeda-Colomer J., Valenciano J., Llopis R. (2014). Justicia social y fair play. Crisis, Cambio Social y Deporte.

[3] Parent S., Fortier K. (2018). Comprehensive overview of the problem of violence against athletes in sport. J. Sport Soc. Issues.

[4] Ulrich R., Pope H.G., Cleret L., Petroczi A., Nepusz T., Schaffer J., Kanayama G., Comstock R.D., Simon P. (2018). Doping in two elite athletics competitions assessed by randomized-response surveys. Sports Med..

[5] Aguilar-Navarro M., Salinero J.J., Muñoz-Guerra J., Plata M.D.M., Del Coso J. (2020). Sport-specific use of doping substances: Analysis of World Anti-Doping Agency Doping Control Tests between 2014 and 2017. Subst. Use Misuse.

[6] Aguilar-Navarro M., Munoz-Guerra J., Plata M., Del Coso J. (2020). Analysis of doping control test results in individual and team sports from 2003 to 2015. J. Sport Health Sci..

[7] Mazzeo F., Altavilla G., D’elia F., Raiola G. (2018). Development of doping in sports: Overview and analysis. J. Phys. Educ. Sport.

[8] Petroczi A., Aidman E. (2009). Measuring explicit attitude toward doping: Review of the psychometric properties of the performance enhancement attitude scale. Psychol. Sport Exerc..

[9] Blank C., Kopp M., Niedermeier M., Schnitzer M., Schobersberger W. (2016). Predictors of doping intentions, susceptibility, and behaviour of elite athletes: A meta-analytic review. SpringerPlus.

[10] Ntoumanis N., Ng J.Y., Barkoukis V., Backhouse S. (2014). Personal and psychosocial predictors of doping use in physical activity settings: A meta-analysis. Sports Med..

[11] Kraus S.J. (1995). Attitudes and the prediction of behavior: A meta-analysis of the empirical literature. Pers. Soc. Psychol. Bull..

[12] Nicholls A.R., Madigan D.J., Duncan L., Hallward L., Lazuras L., Bingham K., Fairs L.R. (2019). Cheater, cheater, pumpkin eater: The Dark Triad, attitudes towards doping, and cheating behaviour among athletes. Eur. J. Sport Sci..

[13] Barkoukis V., Brooke L., Ntoumanis N., Smith B., Gucciardi D.F. (2019). The role of the athletes’ entourage on attitudes to doping. J. Sports Sci..

[14] Engelberg T., Moston S. (2016). Inside the locker room: A qualitative study of coaches’ anti-doping knowledge, beliefs and attitudes. Sport Soc..

[15] Erickson K., McKenna J., Backhouse S.H. (2015). A qualitative analysis of the factors that protect athletes against doping in sport. Psychol. Sport Exerc..

[16] Lazuras L., Barkoukis V., Tsorbatzoudis H. (2015). Toward an integrative model of doping use: An empirical study with adolescent athletes. J. Sport Exerc. Psychol..

[17] Zucchetti G., Candela F., Villosio C. (2015). Psychological and social correlates of doping attitudes among Italian athletes. Int. J. Drug Policy.

[18] Allen J., Taylor J., Dimeo P., Dixon S., Robinson L. (2015). Predicting elite Scottish athletes’ attitudes towards doping: Examining the contribution of achievement goals and motivational climate. J. Sports Sci..

[19] Mudrak J., Slepicka P., Slepickova I. (2018). Sport motivation and doping in adolescent athletes. PLoS ONE.

[20] Wang K., Xu L., Zhang J., Wang J., Sun K. (2020). Relationship between perfectionism and attitudes toward doping in young athletes: The mediating role of autonomous and controlled motivation. Subst. Abuse Treat. Prev. Policy.

[21] Bahrami S., Yousefi B., Kaviani E., Ariapooran S. (2014). The prevalence of energetic drugs use and the role of perfectionism, sensation seeking and physical self-concept in discriminating bodybuilders with positive and negative attitude toward doping. Int. J. Sports Stud..

[22] Bae M., Yoon J., Kang H., Kim T. (2017). Influences of perfectionism and motivational climate on attitudes towards doping among Korean national athletes: A cross sectional study. Subst. Abus. Treat. Prev. Policy.

[23] Ommundsen Y., Roberts G.C., Lemyre P.N., Treasure D. (2003). Perceived motivational climate in male youth soccer: Relations to social–moral functioning, sportspersonship and team norm perceptions. Psychol. Sport Exerc..

[24] Loland S., McNamee M., Morgan W.J. (2015). Fair play. Routledge Handbook of the Philosophy of Sport.

[25] Lucidi F., Zelli A., Mallia L., Nicolais G., Lazuras L., Hagger M.S. (2017). Moral attitudes predict cheating and gamesmanship behaviors among competitive tennis players. Front. Psychol..

[26] Dovovan R.J., Egger G., Kapernick V., Mendoza J. (2002). A conceptual framework for achieving performance enhancing drug compliance in sport. Sports Med..

[27] Gucciardi D.F., Jalleh G., Donovan R.J. (2011). An examination of the Sport Drug Control Model with elite Australian athletes. J. Sci. Med. Sport.

[28] Jalleh G., Donovan R.J., Jobling I. (2014). Predicting attitude towards performance enhancing substance use: A comprehensive test of the Sport Drug Control Model with elite Australian athletes. J. Sci. Med. Sport.

[29] Bandura A., Kurtines W.M., Gewirtz J.L. (1991). Social cognitive theory of moral thought and action. Handbook of Moral Behavior and Development.

[30] Boardley I.D., Smith A.L., Mills J.P., Grix J., Wynne C. (2017). Empathic and self-regulatory processes governing doping behavior. Front. Psychol..

[31] Ring C., Kavussanu M. (2018). The role of self-regulatory efficacy, moral disengagement and guilt on doping likelihood: A social cognitive theory perspective. J. Sports Sci..

[32] Chen Z., Wang D., Wang K., Huang T. (2017). Coaching style and attitudes toward doping in Chinese athletes: The mediating role of moral disengagement. Int. J. Sports Sci. Coach..

[33] Hodge K., Hargreaves E.A., Gerrard D., Lonsdale C. (2013). Psychological mechanisms underlying doping attitudes in sport: Motivation and moral disengagement. J. Sport Exerc. Psychol..

[34] Kavussanu M., Yukhymenko-Lescroart M.A., Elbe A.-M., Hatzigeorgiadis A. (2020). Integrating moral and achievement variables to predict doping likelihood in football: A cross-cultural investigation. Psychol. Sport Exerc..

[35] Ring C., Kavussanu M., Lucidi S., Hurst P. (2019). Effects of personal and situational factors on self-referenced doping likelihood. Psychol. Sport Exerc..

[36] Stanger N., Backhouse S.H. (2020). A multistudy cross-sectional and experimental examination into the interactive effects of moral identity and moral disengagement on doping. J. Sport Exerc. Psychol..

[37] Aquino K., Reed A. (2002). The self-importance of moral identity. J. Pers. Soc. Psychol..

[38] Aquino K., Freeman D., Reed A., Lim V.K.G., Felps W. (2009). Testing a social cognitive model of moral behavior: The interaction of situational factors and moral identity centrality. J. Pers. Soc. Psychol..

[39] Kavussanu M., Stanger N., Ring C. (2015). The effects of moral identity on moral emotion and antisocial behavior in sport. Sport Exerc. Perform. Psychol..

[40] Kavussanu M., Ring C. (2017). Moral identity predicts doping likelihood via moral disengagement and anticipated guilt. J. Sport Exerc. Psychol..

[41] Stamm H., Lamprecht M., Kamber M., Marti B., Mahler N. (2008). The public perception of doping in sport in Switzerland, 1995–2004. J. Sports Sci..

[42] Puchades M., Molina P. (2020). Attitudes towards doping among sport sciences students. Apunt. Educ. Fís. y Deportes.

[43] Vangrunderbeek H., Tolleneer J. (2010). Student attitudes towards doping in sport: Shifting from repression to tolerance?. Int. Rev. Sociol. Sport.

[44] Solberg H.A., Hanstad D.V., Thøring T.A. (2010). Doping in elite sport—do the fans care? Public opinion on the consequences of doping scandals. Int. J. Sport Mark. Spons..

[45] Nicholls A.R., Madigan D.J., Levy A.R. (2017). A confirmatory factor analysis of the performance enhancement attitude scale for adult and adolescent athletes. Psychol. Sport Exerc..

[46] Sukys S., Karanauskiene D. (2020). Adaptation and Validation of the Lithuanian-language version of the Performance Enhancement Attitude Scale (PEAS). J. Phys. Educ. Sport.

[47] Telama R., Naul R., Nupponen H., Rychtecky A., Vuolle P. (2002). Physical Fitness, Sporting Lifestyles, and Olympic Ideals: Cross-Cultural Studies on Youth Sport in Europe.

[48] Majauskiene D. (2013). Manifestation of Olympism and its Cohesion with School Culture and Prosocial Behaviour. Ph.D. Thesis.

[49] Hayes A.F. (2013). Introduction to Mediation, Moderation, and Conditional Process Analysis: A Regression-Based Approach.

[50] Cohen J. (1992). A power primer. Psychol. Bull..

[51] Lee M.J., Whitehead J., Ntoumanis N., Hatzigeorgiadis A. (2008). Relationship among values, achievement orientations, and attitudes in youth sport. J. Sport Exerc. Psychol..

[52] Barkoukis V., Lazuras L., Tsorbatzoudis H., Rodafinos A. (2013). Motivational and social cognitive predictors of doping intentions in elite sports: An integrated approach. Scand. J. Med. Sci. Sports.

[53] Kavussanu M., Hurst P., Yukhymenko-Lescroart M., Galanis E., King A., Hatzigeorgiadis A., Ring C.A. (2020). Moral intervention reduces doping likelihood in UK and Greek athletes: Evidence from a cluster randomized trial. J. Sport Exerc. Psychol..

[54] Ring C., Kavussanu M., Gürpınar B., Whitehead J., Mortimer H. (2020). Basic values predict unethical behavior in sport: The case of athletes’ doping likelihood. Ethics Behav..

[55] Stamm H., Lamprecht M., Kamber M. (2014). Attitudes towards doping—A comparison of elite athletes, performance oriented leisure athletes and general population. Eur. J. Sport Soc..

[56] Bette K.H., Schimank U. (1995). Doping in Hochleistungssport.

[57] García-Grimau E., De la Vega R., De Arce R., Casado A. (2021). Attitudes toward and susceptibility to doping in Spanish elite and national-standard track and field athletes: An examination of the Sport Drug Control Model. Front. Psychol..

[58] Shelley J., Thrower S.N., Petróczi A. (2021). Racing clean in a tainted world: A qualitative exploration of the experiences and views of clean British elite distance runners on doping and anti-doping. Front. Psychol..

